# Adjuvant radiation therapy in stage I seminoma: 20 years of oncologic results

**DOI:** 10.18632/oncotarget.11374

**Published:** 2016-08-18

**Authors:** Francesca De Felice, Daniela Musio, Giovanni Luca Gravina, Francesco Marampon, Vincenzo Tombolini

**Affiliations:** ^1^ Department of Radiotherapy, Policlinico Umberto I, “Sapienza” University of Rome, Rome, Italy; ^2^ Department of Biotechnological and Applied Clinical Sciences, Laboratory of Radiobiology, University of L'Aquila, L'Aquila, Italy; ^3^ Spencer-Lorillard Foundation, Viale Regina Elena, Rome, Italy

**Keywords:** radiotherapy, stage I seminoma, surveillance, chemotherapy, orchiectomy

## Abstract

**Aim:**

To report long term oncologic outcomes after adjuvant radiotherapy (RT) for stage I seminoma.

**Method:**

We reviewed the complete data set for all patients treated at our institute between 1988 and 2005 for stage I seminoma with adjuvant RT after radical orchiectomy.

**Results:**

A total of 85 patients were included. The median follow-up was 15 years. The 20-year overall survival (OS) and relapse free survival (RFS) were 92% and 96.3%, respectively. No severe acute and late complications were recorded. Overall 5.9% of patients had a second unrelated malignancy.

**Conclusion:**

Adjuvant RT is an efficacious and safe treatment in stage I seminoma.

## INTRODUCTION

The vast majority of patients (70% to 80%) diagnosed with seminoma present stage I disease [1].

The primary treatment involves a surgical approach with radical inguinal orchiectomy. Although the cure rates exceed 95%, the optimal post-orchiectomy management remains still debated [1–2]. Radiation therapy (RT), chemotherapy (CHT) and surveillance have evolved separately but currently they are all considered acceptable options with quite similar overall survival (OS) outcome [2–3].

Over the years, RT has been the principal player alongside surgery, due to cells radiation sensitive characteristic. Postoperative RT had a well-documented history as adjuvant treatment of stage I seminoma with an overall relapse rate less than 5% at 5-10 years [4–5]. Acute side effects are usually mild and predictable, whereas incidence of secondary malignancies is difficult to determine.

A randomized trial has investigated single-dose carboplatin as an alternative to RT, and it has been suggested that single course of carboplatin is non-inferior to RT, with similar 5-year relapse rate, but its long effects are still hampered by a short follow-up period [6–7].

With active surveillance, adjuvant treatment is deferred at relapse. The relapse rate is 13% to 24% and salvage therapy is related to recurrence stage disease [8–9].

At our institute, stage I seminoma has for years been routinely treated with adjuvant RT. The purpose of this study is to report long-term follow-up data, in order to analyze related toxicity, incidence of secondary malignancies and oncologic outcomes.

## MATERIALS AND METHODS

### Patients

We reviewed retrospectively the medical data of all consecutive patients treated for stage I seminoma between 1988 and 2005 at our institute. The year 2005 was chosen to allow an adequate follow-up. The study was approved by the institutional review board and the scientific review committee. Data collected included demographics, initial stage disease, treatment details and follow-up.

### Staging system

The extent of primary disease was classified after radical orchiectomy, according to the American Joint Committee on Cancer tumor (T), nodes (N), metastasis (M) Staging System [2]. Stage I disease included pT1-4 N0 M0 and normal serum tumor markers (if marker studies available or performed).

### Treatment

All patients underwent radical inguinal orchiectomy, followed by adjuvant RT. If sperm banking was wished, it was performed either before surgery or RT.

RT was delivered using linear accelerator (4-15 MV). All patients were treated supine, with knee support. Midline and lateral markers were used to align the patient and to prevent lateral rotation. Patients were planned conventionally in a simulator.

Treatment field covered from T10-T11 superiorly to L5-S1 inferiorly. For dog-leg field, the inferior border lay in the top of obturator foramen to include ipsilateral iliac region and surgical scar. Prescribed dose was 30 Gy (2 Gy / fraction) until October 2001, and 25.2 (1.8 Gy / fraction) thereafter.

### Follow-up

Follow-up consisted in complete clinical examination and routine tests, including tumor markers alpha-fetoprotein (α-FP), human chorionic gonadotropin (β-hCG) and lactate dehydrogenase every three months for the first year, every four months for the second year, twice per year for the third year, annually until year ten, and every two years thereafter. Pelvic computed tomography (CT) was performed every 6-12 months for the first 3 years and no routine CT were conducted thereafter.

### Statistical analysis

Standard descriptive statistics were used to evaluate the distribution of each potential factor. Continuous variables were presented as medians and ranges, and dichotomous variables were presented as percentages. Data were compared using non-parametric Fisher exact test for qualitative data and the Wilcoxon test for quantitative data.

Overall survival (OS) and relapse free survival (RFS) were calculated in months from the date of the end of CRT to the first event, including date of the last follow-up or death (OS), and/or relapse (RFS). Second malignancy was defined as the diagnosis of a new tumor lesion detected at least 5 years after the end of RT. OS and RFS were estimated according to Kaplan-Meier method. Statistical analysis was performed using RStudio-0.98.1091 software.

## RESULTS

### Patient and treatment characteristics

Overall 85 patients were included in the study. Patients and tumor characteristics are shown in Table 1. Preoperative serum tumor markers were recorded in 74.1% of patients (*n* = 67) and levels were in the normal range before RT. Median age at diagnosis was 36 years (range 22 – 77). The vast majority of patients (*n* = 58; 68.2%) had RT to abdominal and ipsilateral pelvic lymph nodes. Treatment details are given in Table 2.

**Table 1:** Patients and tumor characteristics

Characteristic	Patient (%)
Age (years)	
median (range)	36 (22 -72)
Primary tumor (T)	
1	55 (64.7)
2	25 (29.4)
3	3 (3.5)
4	2 (2.4)
Regional lymph nodes (N)	
negative	85 (100)
positive	0 (0)
Serum tumor markers (S)	
0	
x	
Stage	
IA	55 (64.7)
IB	30 (35.3)

**Table 2:** Radiation therapy treatment details

Characteristic	Patient (%)
Treatment field	
dog-leg*	58 (68.2)
T10-11 to L5-S1	27 (31.8)
RT machine	
linear accelerator	85 (100)
Total dose (Gy)	
30	58 (68.2)
25.2	27 (31.8)

* inferior border: top of obturator foramen to include ipsilateral iliac region and surgical scar

### Clinical outcomes

At the date of analysis, 4 patients (4.7 %) were dead, 76 patients (89.4 %) were alive and 5 patients (5.9 %) were lost for follow-up. No patient had died of seminoma cancer. Major causes of death were related to a second carcinoma (*n* = 2) or to other coexisting medical condition (*n* = 2). The median follow-up was 15 years (range 7 – 26).

**Figure 1:**
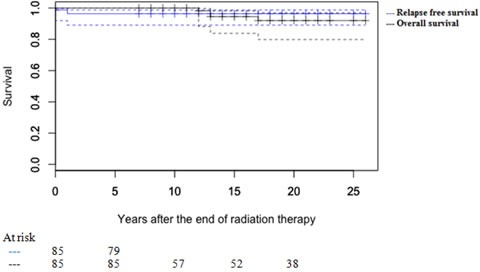
Kaplan Meier curves of overall survival (OS) and relapse free survival (RFS)

The 10- and 20-year OS rates for the entire group of patients were 100% and 92% (95% CI: 0.79 - 0.97), respectively. The 10- and 20-year RFS were estimated at 96.3% (95% CI 0.89 - 0.99) (Figure 1). All relapses (*n* = 3; 3.5%) occurred in those patients with pT2 disease and within 2 years after RT. Treatment was chemotherapy, based on bleomycin, etoposide and cisplatin (BEP) regimen in case of pelvic (iliac nodes, *n* = 2) and distant relapse (mediastinal mass, *n* = 1). Of the patients who relapsed, all are still alive and disease free.

### Second malignancies

Overall 6 patients (7.1%) developed a second cancer and all of these patients were treated with “dog-leg” field. There have been 1 (1.2%) controlateral testicular tumor, and 5 (5.9%) unrelated tumors, including 1 leukemia and 4 solid carcinomas (kidney, *n* = 1; lung, *n* =1; pancreas, n = 1, neurofibrosarcoma, *n* = 1). Median time to secondary malignancies was 18 years (range, 13-26) and most of them (*n* = 4; 66.7%) were observed beyond 15 years after the end of RT.

### Acute and late toxicity

Details are shown in Table 3. Overall data on acute and late toxicity were not significantly different between RT treatment field.

**Table 3:** Incidence of acute and late toxicity

	Patient (N)			
Toxicity	Total	T10-11 to L5-S1	Dog-leg	p-value
*Acute*				
Blood-bone marrow	0	0	0	1.0
Neutrofilis-granulocytes	0	0	0	
Gastrointestinal				
Diarrhea	15	4	11	0.65
Nausea	41	18	23	0.2
Vomiting	3	1	2	0.77
Renal-genitourinary				
Dysuria	0	0	0	1.0
Urinary frequency	0	0	0	
*Late*				
Infertility	7	2	5	0.83
Cardiovascular disease	1	0	1	0.66
Gastrointestinal disease	3	1	2	0.77
Second malignancy	6	0	6	0.11

Among the 85 patients, there were no severe (> grade 2) acute complications. The main acute symptoms were nausea (*n* = 41; 48.2%), associated with vomiting in 3 cases only, and diarrhoea (*n* = 15; 17.6%). No hematologic or renal acute toxicity has been observed.

Late treatment related toxicity included infertility (*n* = 7; 8.2%), cardiovascular disease (*n* = 1; 1.2%), poliposis of the gastrointestinal tract (*n* = 2; 2.4%) and peptic ulcer disease (*n* = 1; 1.2%). No osteonecrosis was recorded.

## DISCUSSION AND CONCLUSIONS

Our results demonstrate an excellent prognosis in stage I seminoma patients after adjuvant RT. Over the time period of the study, RT treatment policies evolved to reflect the tendency toward lower doses and smaller volumes. However, the vast majority of patients reported a satisfactory quality of life (QoL), without severe complications, including cancer/treatment related death. Second cancer remains the major late treatment related morbidity, with a small risk (5.9%) of unrelated secondary malignant tumors.

These results are consistent with those reported in literature. In general it is assumed an actuarial risk of second malignancies from 5% to 8% [10]. The largest study of second cancers in testicular cancer survivors was presented by Travis et al [11]. Results showed a decreasing risk for second cancers with increasing age at diagnosis. Survivors were at statistically significant increased risk of solid tumors for at least 35 years after the end of RT and at 20 years after RT the cancer risk was double over general population [11].

Surgery followed by RT has been known for decades to be the cornerstone of stage I seminoma treatment. In the recent years, adjuvant RT has been removed from most clinical guidelines principally due to the discover of the induced secondary cancers. Several alternatives to adjuvant RT have been proposed and current treatment options include active surveillance or adjuvant CHT in the form of carboplatin.

Treatment-related effects are extremely important in stage I seminoma, due to its exceptionally high cure rate and its peak of young age diagnosis. Long term cancer survivors have a life expectancy of 40-60 years following to successful treatment. Late toxicities, especially second malignancies, are the most serious effect from seminoma RT treatment. But it should be noted that there are limited experience that analyse second malignancy risk in patients treated with alternatives to adjuvant RT.

Active surveillance strategy is based on regular follow-up, including markers, chest radiograph and abdominal/pelvic CT scan, to detect any recurrence at an early stage. Even if patients experience a certain level of anxiety due to fear of recurrence, surveillance management can avoid further treatment. Warde et al. identified two risk factors, including size of primary tumor and rete testis involvement, as significant prognostic factors for recurrence in stage I seminoma [12]. Despite the difficulties in discerning rete testis invasion, as well as in defining tumor size due to lesion multifocality, it has been possible to structure a prognostic nomogram model, using these two risk factors. Recurrence risk was estimated over 30% in case of both risk factors, whereas it was only half of that in case of only one or neither risk factor [13]. However, overall post resection relapse rate is approximately 20 % and patients who relapsed will receive more anticancer treatment than is needed in adjuvant setting [14].

Moreover, considering the young age of many patients and the non-specificity of tumor markers, surveillance strategy involves multiple serial abdominal/pelvic CT over a long time period. For instance, the NCCN surveillance protocol recommends an abdominal/pelvic CT at 3,6, and 12 months during the first 1 year of follow-up, every 6-12 months during the second and third year and annually thereafter [2]. Consequently the routinely use of CT scan exposes patients to low-level of ionising radiations that are associated at some risk of induced cancer. It has been estimated that the relative risk of a secondary malignancy with surveillance program compared to a single CT after surgery is approximately 15.2 [15]. Anyway, definitive data on potential late effects, in term of second cancer risk related to surveillance strategy, as well as comparative data with RT long term toxicity are still lacking in literature and further studies are necessary.

Adjuvant CHT represents another valid approach. Adjuvant carboplatin is the main option that has been explored over the years. The MRC TE19/EORTC trial was the first study that describes the use of carboplatin in adjuvant setting [6]. Patients were randomized either RT either carboplatin single cycle. Update results confirmed the non inferiority of CHT versus RT in term of RFS at 5 years (94.7 % versus 96%, respectively) [7]. However the trial was originally powered to exclude a 3% absolute relapse difference in any pair-wise comparison (30 Gy versus 20 Gy versus carboplatin); RFR absolute difference at 5 years was estimated 1.34% (90% CI, - 0.7% to 3.5%), thus the primary outcome measure was not completely achieved.

However, independently of post-orchiectomy management, young adult patients continue to be treated, therefore it is important to evaluate future risks. It is evident that effects on QoL have become one of the important factors influencing patients and clinicians in choosing the best therapy approach. Although an increased risk of second cancer is recognised after RT, its impact after CHT or surveillance remains controversial. Adjuvant RT might represent an overtreatment, but improved outcomes after CHT or surveillance are needed.

This study had several limitations. Our knowledge about late effects is based on treatment that was administered over 10 years ago and thus it implicated a retrospective nature of the study. However, this is the first long-term follow-up analysis showing oncologic outcomes at 20 years. Only few patients were lost at follow up and therefore results were not negatively affected, but a detailed assessment of fertility and gonadal status was not possible, due to missing data in a significant proportion of reports.

Cure rate is very high with either surveillance or adjuvant treatment following radical orchiectomy.

The potential of relapse using surveillance, on one hand, and the potential of late toxic effects especially from adjuvant treatment, on the other hand, should be carefully considered. It should be recommend patient autonomy, after unbiased information. Surely, a more precise risk classifications and a matured data on both adjuvant CHT and surveillance long-term toxicity could improve quality of clinical advise.

Stage I seminoma patients have an excellent prognosis. Our results confirmed RT efficacy in this setting of patients. The minimal risk of relapse and the low incidence of late effects justify adjuvant RT. Waiting for surveillance and CHT mature long term toxicity data, a long-time horizon in decision-making should be recommended.
